# Bioassay-guided fractionation, phospholipase A_2_-inhibitory activity and structure elucidation of compounds from leaves of *Schumanniophyton magnificum*

**DOI:** 10.1080/13880209.2020.1839510

**Published:** 2020-11-09

**Authors:** Parker Elijah Joshua, Chizoba Joy Anosike, Rita Onyekachukwu Asomadu, Daniel Emmanuel Ekpo, Emmanuel Nnaemeka Uhuo, Okwesili Fred Chiletugo Nwodo

**Affiliations:** a Department of Biochemistry, Faculty of Biological Sciences, University of Nigeria, Nsukka, Enugu State, Nigeria; b Department of Biochemistry, Michael Okpara University of Agriculture, Umudike, Abia State, Nigeria; c Department of Biochemistry, Faculty of Medical, Pharmaceutical and Health Sciences, University of Mkar, Mkar, Benue State, Nigeria

**Keywords:** Rubiaceae, anti-inflammatory, *Bacillus cereus*, enzyme inhibition, hexadecanoic acid, ethyl ester, 3-ethylhexane, 14-Methyl pentadecanoic acid methyl ester, gas chromatography

## Abstract

**Context:**

*Schumanniophyton magnificum* Harms (Rubiaceae) is used traditionally in Nigeria for the treatment of snake bites. Snake venom contains phospholipase A_2_ (PLA_2_) which plays a key role in causing inflammation and pain.

**Objective:**

To assess the anti-inflammatory effect of the methanol extract of *Schumanniophyton magnificum* (MESM) leaves through the inhibition of PLA_2_ and investigate the compounds responsible for the effect.

**Materials and methods:**

PLA_2_-inhibitory activity of MESM was assessed at concentrations of 0.1–0.8 mg/mL using human red blood cells as substrate. Prednisolone was used as the standard control. MESM was subsequently partitioned using *n*-hexane, dichloromethane, ethyl acetate and aqueous-methanol (90:10 v/v), after which PLA_2_-inhibitory activity of the partitions was determined. The best partition was subjected to chromatographic techniques and the fractions obtained were assessed for PLA_2_ inhibition at 0.4 mg/mL. Compounds in the most active fraction were determined using Fourier-transform infrared spectroscopy **(**FTIR) and gas chromatography-mass spectrometry (GC-MS).

**Results:**

MESM significantly inhibited PLA_2_ activity at 0.8 mg/mL (44.253%) compared to prednisolone (35.207%). *n*-Hexane partition (SMP1) proved more active with inhibition of 55.870% observed at 0.1 mg/mL. Fraction 1 (SMF1) showed the highest PLA_2_-inhibitory activity of 58.117%. FTIR studies revealed the presence of some functional groups in SMF1, and GC-MS confirmed the presence of 9 compounds which are first reported in this plant. Hexadecanoic acid, ethyl ester was identified as the major compound (24.906%).

**Discussion and conclusions:**

The PLA_2_-inhibitory activity of MESM suggests that its compounds may be explored further in monitoring anti-inflammatory genes affected by the venoms.

## Introduction

Inflammation, which is the response of the immune system to harmful stimuli, such as pathogens, damaged cells, irradiation or toxic compounds, acts by removing the injurious stimuli and initiating the healing process (Chen et al. 2018). However, sustained inflammation can be detrimental to the host as it contributes to the pathogenesis of many diseases. Inflammation is mediated by some chemical substances, such as serotonin, histamine, leukotrienes, and prostaglandins (Abdulkhaleq et al. 2018). Prostaglandins are synthesized as a result of the cleaving of arachidonic acid from membrane phospholipids through the action of phospholipase A_2_ (PLA_2_).

Much interest has therefore been focussed on PLA_2_ in the context of inflammation. Although, non-steroidal anti-inflammatory drugs (NSAIDs) are still widely employed as analgesics and to relief inflammatory conditions (Fokunang et al. 2017), their adverse effects pose some limitations in their use. Hence, there is a need to continuously search for more effective and safer alternatives. With respect to this, medicinal herbs are important globally, and the quest for novel plants with improved biological properties is a continuous exercise.

*Schumanniophyton magnificum* Harms (Rubiaceae), commonly known as ‘mgba mmiri’ in Ibo language of Nigeria, is widely used to treat various diseases in African ethnomedicine. The decoction of the stem bark is used in Cameroon to treat dysentery and as a lotion after circumcision (Okogun et al. 1983). Its stem bark is also used in Congo as an antiseptic for the treatment of gonorrhoea and ulcers. The stem bark extracts are also effective against *Plasmodium falciparum* (Bickii et al. 2007), and ability of the stem bark aqueous extract to improve fertility has also been demonstrated (Bend et al. 2018). *S. magnificum* is used as a snake bite remedy and as snake repellent in Nigeria. The protective effects of the stem bark extract against snake venom have been demonstrated (Akunyili and Akubue 1986; Houghton et al. 1992). However, while there are claims on its use traditionally for the treatment of snake bite and relieve of pain, there are no scientific reports on the use of its leaves for this purpose. Since local inflammation and pain are important features of snakebite envenomation due to increase in the activities of PLA_2_ present in the venom (Tonello and Rigoni 2017), and *S. magnificum* has potential in inhibiting PLA_2_ (Mohanapriya et al. 2017; Sajon et al. 2017), this study seeks to investigate and establish a scientific report on the anti-inflammatory effects of *S. magnificum* leaves *via* the inhibition of PLA_2_ activity. It also seeks to investigate the nature of compounds responsible for the effects.

## Materials and methods

### Blood samples

Blood samples were collected from healthy donors with their informed consent in line with the Declaration of Helsinki. Participants were free from any drug treatment for at least two weeks before blood collection. Ethical approval was obtained from the Institutional Review Board (Ethics and Biosafety Committee, Faculty of Biological Sciences, University of Nigeria, Nsukka, Nigeria) with approval number: UNN/FBS/EC/1008.

### Microorganism

*Bacillus cereus*, which was used as a source of PLA_2_ was obtained from the Department of Microbiology, University of Nigeria, Nsukka, Nigeria.

### Collection of plant material

The fresh leaves of *S. magnificum* were collected in July 2017 from Ukehe town in Igbo Etiti Local Government of Enugu State, Nigeria. The leaves were identified and authenticated by Mr. Alfred Ozioko of International Centre for Ethnomedicine and Drug Development (InterCEDD) Nsukka, Enugu State, Nigeria where the voucher number Intercedd/837 was deposited for reference purposes. The leaves were dried under room temperature and pulverized to coarse powder using a milling machine.

### Extraction of plant material

A weighed quantity (1974.92 g) of the ground leaves was extracted with methanol by cold maceration for 48 h at room temperature (26–28 °C). The macerate was filtered using Whatman No. 1 filter paper and concentrated using a vacuum rotary evaporator at 40–60 °C. The obtained methanol extract of *Schumanniophyton magnificum* (MESM) stored in an air-tight plastic container in the refrigerator (4 °C) was used for the study.

### *Partitioning of the methanol extract* Schumanniophyton magnificum

MESM was partitioned using *n*-hexane, dichloromethane, ethyl acetate and aqueous-methanol (90:10 v/v) according to their polarity as described by Rajbir et al. (2008). MESM (10 g) was dissolved in 250 mL of aqueous-methanol and poured into a separating funnel, after which 250 mL of *n*-hexane was added, shaken vigorously and allowed to stand. This led to the development of two layers; upper and lower layers. The upper layer was collected and designated the *n*-hexane partition (SMP1). The lower layer was poured back to the funnel and re-washed with *n*-hexane. Dichloromethane was thereafter introduced into the aqueous portion, and was treated as *n*-hexane. The lower layer represents the dichloromethane partition (SMP2). After re-washing with dichloromethane, ethyl acetate was introduced, treated likewise and the ethyl acetate partition (SMP3) was obtained. The last portion was the aqueous-methanol partition (SMP4). In all, 30 g of MESM was partitioned. The partitions were subsequently concentrated using a rotary evaporator.

### Phytochemical screening

The phytochemical analysis of MESM and its partitions were carried out according to the methods of Harborne (1998) and Trease and Evans (2002).

### Phospholipase A_2_ activity

The effect of MESM and its partitions on PLA_2_ activity was determined using the method of Vane (1971) as modified by Oyekachukwu et al. (2017). Fresh human blood samples were centrifuged at 3000 rpm for 10 min after which the supernatant (plasma) was discarded. The red cells were thereafter washed three times with equal volume of normal saline and reconstituted as a 40% (v/v) suspension with normal saline. This served as the substrate for PLA_2_. Bacterial enzyme preparation was gotten from *Bacillus cereus* strain culture. The organism was cultured in MaCconkey agar for 72 h and the culture centrifuged with normal saline at 3000 rpm for 10 min. The bacterial cells settled at the bottom of the centrifuge tube while the enzyme was contained in the supernatant. Aliquots (0.1 mL) of re-suspended erythrocytes (substrate), normal saline (1.2 mL), 2 mM calcium chloride (0.2 mL), 1 mL varying concentration of MESM and partitions (0.1, 0.2, 0.4, 0.6 and 0.8 mg/mL) in 3% Tween 80 (dissolved in normal saline) and 0.2 mL of the enzyme preparation were incubated at 37 °C for 1 h. Control tubes contained 0.1 mL of the erythrocytes, 1.2 mL normal saline, 0.2 mL calcium chloride, 1 mL 3% Tween 80 and 0.2 mL of the enzyme preparation. The blanks contained everything in the test except the enzyme. After incubation, the reaction mixtures were centrifuged at 3000 *g* for 10 min and the absorption of the supernatants read against the blank at 418 nm. A known inhibitor of the enzyme (prednisolone; 0.4 mg/mL), was used as the standard control. The percentage inhibition of enzyme activity was calculated relative to control thus:

$$\text{\% Enzyme activity }=\;\frac{\text{Absorbance of test}}{\text{Absorbance of control}}\;\times 100$$

% inhibition of enzyme activity = 100 − % Enzyme activity

### Thin-layer chromatography

The most potent partition (SMP1) was spotted on thin-layer chromatographic plates pre-coated with silica-gel (Merck, silica gel 60 F_254_). The plates were developed in chromatographic tanks with toluene:ethyl acetate (80:20) as the solvent system which gave about 6–8 visible bands when viewed under ultraviolet (UV) light. The plates were allowed to stay for elution to be achieved. SMP1 was further purified using column chromatography.

### Column chromatography

Silica gel (100 g) of mesh size 60–120 were mixed with 250 mL of the eluent, toluene: ethyl acetate (80:20) and stirred for 15 min to achieve a uniform consistency. The column was subsequently packed with the silica gel using a funnel, after which SMP1 was introduced by pouring through the wall of the column. This was subsequently eluted at 5 mL volume interval. In all, 80 fractions of 5 mL each were collected. The fractions were spotted on TLC plate and their retention factor (R_f_) value calculated. The fractions with similar R_f_ value were pooled together. Four sub fractions (SMF1, SMF2, SMF3 and SMF4) were obtained in all. These fractions were also subjected to PLA_2_ activity at 0.4 mg/mL.

### Fourier-transform infrared spectroscopy (FTIR)

The FTIR of the most potent fraction (SMF1) was carried out using FTIR-8400S spectrophotometer (Shimadzu model, Kyoto, Japan). SMF1 was used in a form of a thin film, held in between two potassium bromide discs. The liquid paste was dropped on each disc, after which they spread into a thin film. The disc was then mounted in the FTIR spectrometer in the range 1.20 × 1013 − 1.20 × 1014 Hz within the infrared region of the electromagnetic spectrum. Absorption was written in terms of wave numbers (units; cm^−1^).

### Gas chromatography-mass spectrometry (GC-MS)

Chemical composition of SMF1 was determined by GC/MS-QP-2010 plus Ultra (Shimadzu, Kyoto, Japan) using a DB-5 MS fused silica capillary column (30 × 0.25 m internal diameter, film thickness 0.25 µm). An electron ionization system with ionization energy of 70 eV was used. Carrier gas was helium gas, flow rate was 1.2 mL/min. Injector and MS transfer line temperature were set at 260 and 270 °C, respectively. Oven temperature was initially maintained for 2 min, and then increased to 210 °C at a rate of 8 °C/min to 280 °C; hold time was 10 min. Samples were completely dissolved in absolute ethanol and 0.3 µL was injected through auto-sampler in the split mode, split ratio was 1:100. Relative percentage of each constituent was expressed as percentages by peak area normalization. Each component was identified based on its column retention time (RT) relative to computer-based matching of mass spectra with those of standards: National Institute of Standards and Technology (NIST) and Wiley libraries for GC-MS system.

### Statistical analysis

Data obtained were analysed using one-way analysis of variance (ANOVA) and subjected to Duncan *post hoc* test for multiple comparisons. Results are presented as mean ± standard deviation and differences between means were significant at 95% confidence interval.

## Results

### Phytochemical composition

The phytochemical screening of MESM and its partitions revealed varying secondary metabolites as shown in Table 1.

**Table 1.** Phytochemical composition of methanol extract and partitions of *Schumanniophyton magnificum*.

Phytochemicals	MESM	SMP1	SMP2	SMP3	SMP4
Alkaloids	++	++	++	+	+
Terpenoids	++	++	+++	ND	ND
Phenols	++	+	++	+++	+
Saponins	++	ND	ND	ND	+
Flavonoids	+	+	+	++	++
Tannins	+	ND	++	+++	ND
Carbohydrates	+++	+	+	+	+
Proteins	++	ND	ND	ND	ND
Glycosides	ND	++	+	+	+
Reducing sugars	ND	+	ND	+	+
Steroids	ND	++	+++	ND	ND

+ Slightly present, ++ moderately present, +++ highly present.

ND: not detected; MESM: methanol extract of *Schumanniophyton magnificum*; SMP1: *Schumanniophyton magnificum* partition 1 (*n*-hexane partition); SMP2: *Schumanniophyton magnificum* partition 2 (dichloromethane partition); SMP3: *Schumanniophyton magnificum* partition 3 (ethyl acetate partition); SMP4: *Schumanniophyton magnificum* partition 4 (aqueous-methanol partition).

### *Effect of methanol extract of* Schumanniophyton magnificum *on phospholipase A_2_ activity*

As shown in Table 2, MESM significantly (*p* < 0.05) inhibited PLA_2_ activity at all the concentrations compared to control. The highest inhibitory activity (44.253%) was observed at 0.8 mg/mL.

**Table 2.** Effect of methanol extract of *Schumanniophyton magnificum* on phospholipase A_2_ activity.

Treatment	Concentration (mg/mL)	Absorbance	Percentage inhibition (%)
Control		0.534	0.000 ± 0.000^a^
MESM	0.1	0.363	32.083 ± 0.110^b^
	0.2	0.351	34.207 ± 0.110^c^
	0.4	0.342	36.020 ± 0.104^e^
	0.6	0.316	40.760 ± 0.285^f^
	0.8	0.298	44.253 ± 0.110^g^
Prednisolone	0.4	0.346	35.207 ± 0.185^d^

Results are presented as mean ± SD, *n* = 3. Mean values with different letters as superscripts are considered significant at *p* < 0.05

MESM: methanol extract of *Schumanniophyton magnificum*.

### *Effect of* Schumanniophyton magnificum *partitions on phospholipase A_2_ activity*

Table 3 shows the effect of the various partitions of MESM on PLA_2_ activity. The partitions exhibited the highest inhibitory effect at 0.1 mg/mL, with SMP1 showing the highest inhibition of 55.870%.

**Table 3.** Effect of partitions of *Schumanniophyton magnificum* on phospholipase A_2_ activity.

Absorbance and percentage (%) inhibition of PLA_2_ activity							
	Control	0.1 mg/mL	0.2 mg/mL	0.4 mg/mL	0.6 mg/mL	0.8 mg/mL	Prednisolone (0.4 mg/mL)
SMP1	0.534 (0.000 ± 0.000^a^)	0.236 (55.870 ± 0.285^g^)	0.242 (54.743 ± 0.110^f^)	0.250 (53.123 ± 0.214^e^)	0.258 (51.750 ± 0.104^d^)	0.264 (50.560 ± 0.190^c^)	0.346 (35.207 ± 0.185^b^)
SMP2	0.534 (0.000 ± 0.000^a^)	0.410 (23.157 ± 0.110^f^)	0.416 (22.160 ± 0.104^e^)	0.418 (21.660 ± 0.104^d^)	0.436 (18.413 ± 0.290^c^)	0.442 (17.290 ± 0.392^b^)	0.346 (35.207 ± 0.185^g^)
SMP3	0.534 (0.000 ± 0.000^a^)	0.396 (25.843 ± 0.185^f^)	0.398 (25.407 ± 0.110^e^)	0.402 (24.783 ± 0.110^d^)	0.408 (23.600 ± 0.000^c^)	0.416 (22.097 ± 0.185^b^)	0.346 (35.207 ± 0.185^g^)
SMP4	0.534 (0.000 ± 0.000^a^)	0.238 (55.493 ± 0.110^g^)	0.250 (53.243 ± 0.110^f^)	0.254 (52.433 ± 0.185^e^)	0.266 (50.250 ± 0.285^d^)	0.274 (48.753 ± 0.110^c^)	0.346 (35.207 ± 0.185^b^)

Results are presented as mean ± SD, *n* = 3. Mean values with different letters as superscripts across the rows are considered significant at *p* < 0.05.

Percentage inhibitions of PLA_2_ activity are in parenthesis.

SMP1: *Schumanniophyton magnificum* partition 1 (*n*-hexane partition); SMP2: *Schumanniophyton magnificum* partition 2 (dichloromethane partition); SMP3: *Schumanniophyton magnificum* partition 3 (ethyl acetate partition); SMP4: *Schumanniophyton magnificum* partition 4 (aqueous-methanol partition).

### *Effect of* Schumanniophyton magnificum *fractions on phospholipase A_2_ activity*

SMF1 had the highest inhibitory effect of 58.117%, followed by SMF3, SMF4 and SMF2 with inhibition of 54.807, 53.620 and 52.373%, respectively (Table 4).

**Table 4.** Effect of fractions of *Schumanniophyton magnificum* on phospholipase A_2_ activity.

Treatment	Concentration (mg/mL)	Absorbance	Percentage inhibition (%)
Control		0.534	0.000 ± 0.000^a^
SMF1	0.4	0.224	58.117 ± 0.964^f^
SMF2	0.4	0.254	52.373 ± 0.657^c^
SMF3	0.4	0.241	54.807 ± 0.571^e^
SMF4	0.4	0.248	53.620 ± 0.285^d^
Prednisolone	0.4	0.346	35.207 ± 0.185^b^

Results are presented as mean ± SD, *n* = 3. Mean values with different letters as superscripts are considered significant at *p* < 0.05.

SMF1: *Schumanniophyton magnificum* fraction 1; SMF2: *Schumanniophyton magnificum* fraction 2; SMF3: *Schumanniophyton magnificum* fraction 3; SMF4: *Schumanniophyton magnificum* fraction 4.

### *FTIR frequency range and functional groups present in* Schumanniophyton magnificum *fraction 1(SMFI)*

**Figure 1.**
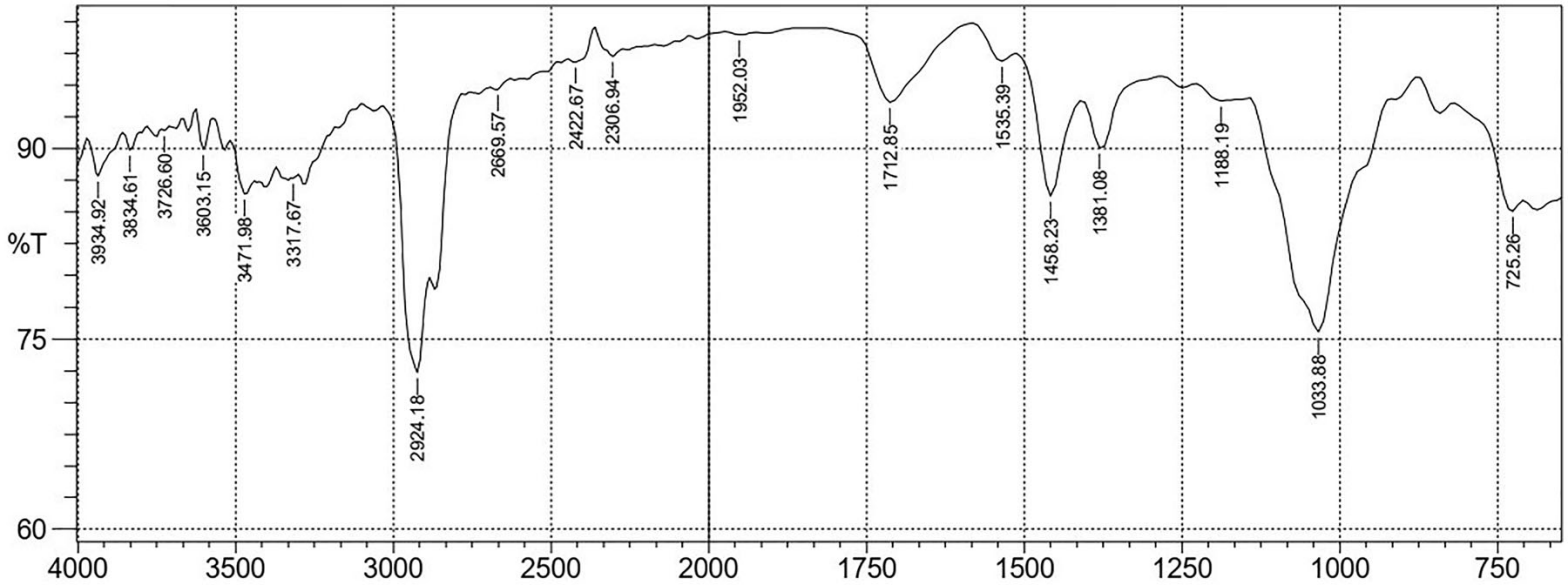
FTIR spectra of *Schumanniophyton magnificum* fraction 1.

As shown in Figure 1, SMF1 spectra revealed strong absorption peak at a frequency of 2924.18 and 1033.88 cm^−1^ assigned to C–H of alkanes and C–O of phenols, respectively. Other peaks indicated the presence of amines, phenols, carboxylic acid, nitrites, esters, aldehydes, ketones, and alkenes (Table 5).

**Table 5.** FTIR frequency range and functional groups present in *Schumanniophyton magnificum* fraction 1.

Wave number (cm^−1^)	Functional groups (vibration mode)	Possible compounds present
3834.61; 3934.92	N–H str. (w)	Amines
3603.15; 3726.60	O–H str. (m)	Alcohols, phenols
3317.67; 3471.98	N–H str. (m)	Amines
2924.18	C–H str. (s)	Alkanes
2669.57	O–H str. (w)	Carboxylic acid
2306.94, 2422.67	NΞH str. (w)	Nitrites
1952.03	C = C=C str. (w)	Alkenes
1712.85	C = O str. (m)	Esters, aldehydes and ketones
1535.39	C = C str. (w)	Esters, aldehydes and ketones
1381.08, 1458.23	C–H deformation (m)	Alkanes
1188.19	C–O str. (w)	Phenols and esters
1033.88	C–O rocking (s)	phenols
725.26	C–H deformation (m)	Alkenes

Key: w: weak; m: medium; s: strong; str.: stretching.

### *Biologically active chemical compounds in* Schumanniophyton magnificum *fraction 1*

**Figure 2.**
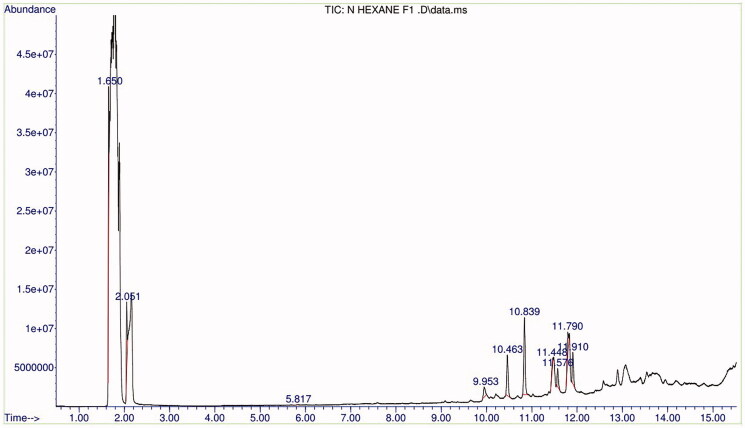
Chromatogram of *Schumanniophyton magnificum* fraction 1.

The compounds present in SMF1were identified by GC-MS analysis. Spectrogram showing the peak identities of the compound is shown in Figure 2. The nine compounds identified with their RT, molecular formula (MF), concentration (%) are presented in Table 6.

**Table 6.** Biologically active chemical compounds of *Schumanniophyton magnificum* fraction 1.

Name of compounds (molecular formula)	Chemical structure	Retention time (min)	%
3-Ethylhexane (C_8_H_18_)	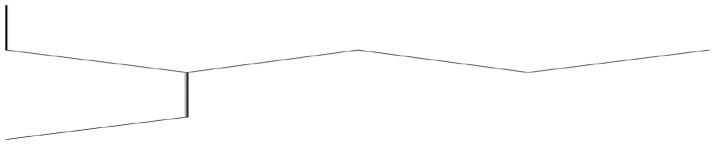	1.652	21.669
Toluene (C_7_H_8_)	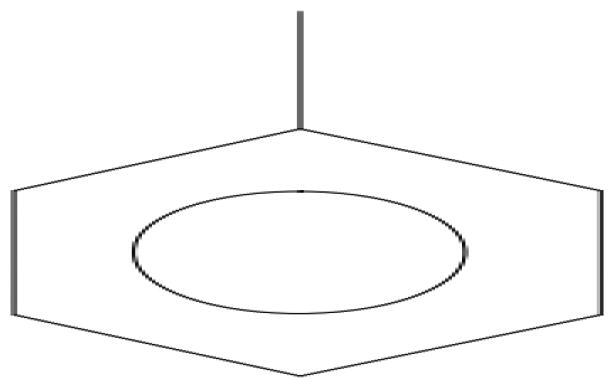	2.051	11.977
2,6,10-Trimethyl tetradecane (C_17_H_36_)	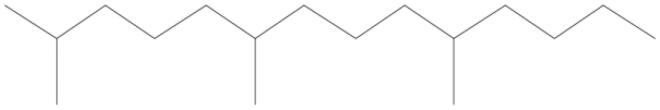	5.817	0.086
(*Z*)-2-(9-Octadecenyloxy)ethanol (C_20_H_40_O_2_)	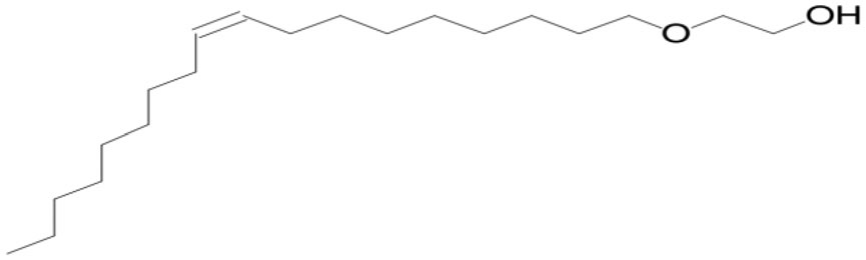	9.953	4.028
14-Methyl pentadecanoic acid methyl ester (C_17_H_34_O_2_)	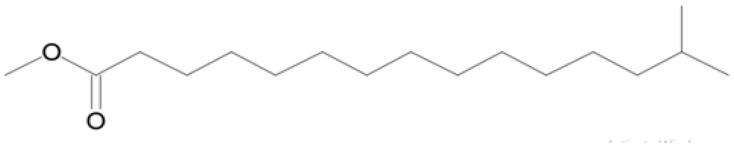	10.465	13.351
Hexadecanoic acid, ethyl ester (C_18_H_36_O_2_)	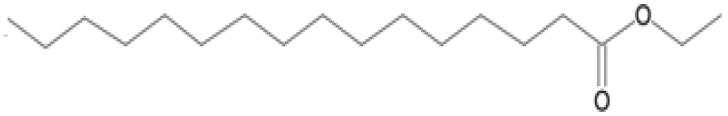	10.838	24.906
Linoleic acid ethyl ester (C_20_H_36_O_2_)	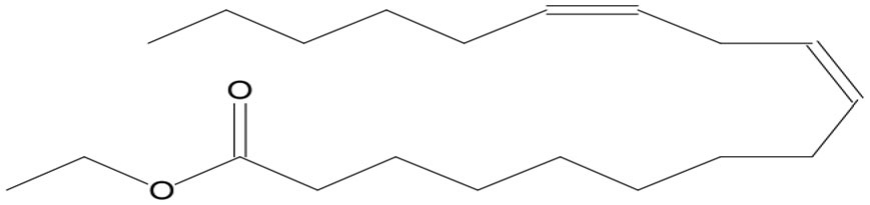	11.448	2.942
9-Methyl heptadecanoic acid methyl ester (C_19_H_38_O_2_)	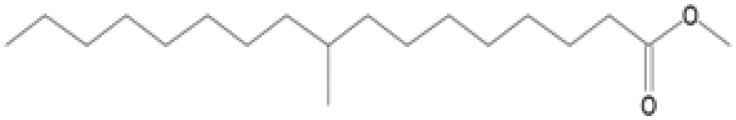	11.577	5.300
Linoleic acid ethyl ester (C_20_H_36_O_2_)	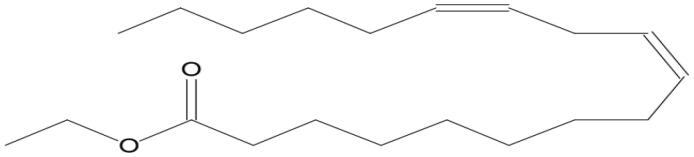	11.791	7.485
Octadecanoic acid, ethyl ester (C_20_H_40_O_2_)	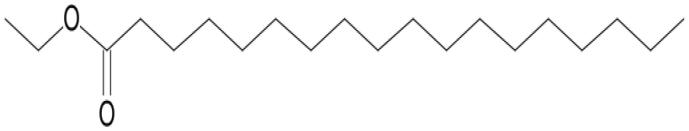	11.910	8.256

## Discussion

Natural products obtained from medicinal plants in the form of pure compounds or standardized extracts enable the discovery of novel drug leads due to the unmatched availability of diverse chemical compounds (Mustafa et al. 2017). There are several phytochemicals from plants which are better alternatives for the treatment of diseases due to reduced adverse effects in relation to chemically synthesized drugs.

The primary metabolites present in MESM shows the presence of alkaloids, terpenoids, phenols, saponins, flavonoids, tannins, carbohydrates and proteins. Alkaloids, phenols and flavonoids present in MESM and its partitions were also obtained from the stem bark extract of *S. magnificum* as confirmed by Tchouya et al. (2014). Reducing sugars were also absent in the stem bark extract of *S. magnificum* (Tchouya et al. 2014) in line with this study. The presence of these diverse phytoconstituents in the leaves of *S. magnificum* is indicative of its diverse biological activities. Alkaloids produce antimicrobial, analgesic, antifungal, anti-inflammatory, anti-fibrogenic and antispasmodic effects (Ibraheem and Maimako 2014; Renuga and Krishnakumari 2015). Different chromone alkaloids have been isolated from the stem bark (Akunyili and Akubue 1986) and root bark of *S. magnificum* (Houghton and Hairong 1985). Terpenoids possess anti-inflammatory, antimalaria, anticancer, sedative, insecticidal or cytotoxic activity. Phenolic compounds possess anti-inflammatory properties (Zhu et al. 2018); flavonoids and tannins also have anti-inflammatory properties (Owolabi et al. 2018). The anti-inflammatory properties of flavonoids are due to their ability to inhibit enzymes involved in the production of chemical mediators of inflammation, such as PLA_2_ (Iwuala et al. 2015; Owolabi et al. 2018). They also exert their effects as antioxidants, free radical scavengers, and chelators of divalent cation (Middleton et al. 2000). Therapeutic properties of saponins include antimicrobial effect (Habu and Ibeh 2015), antioxidant (Shirwaikar et al. 2003), and anticancer activities, stimulation of antibody production and anti-inflammatory properties (Abubakar et al. 2015). Carbohydrates are useful as they supply energy to the cells, spare protein as a source of energy; contribute to fat metabolism and aid assimilation of minerals (Eze and Obinwa 2014). The presence of protein indicates that the leaves could serve as a vital tool for growth and development, serve as an enzymatic catalyst and cell response mediators (Ojo et al. 2014).

PLA_2_ hydrolyses fatty acids at the *sn*-2 position of membrane phospholipids releasing lysophospholipids and arachidonic acid (Cedro et al. 2018). Arachidonic acid in turn leads to the production of eicosanoids such as prostaglandins, thromboxanes and leukotrienes (Yui et al. 2015), which are mediators of inflammatory effect. MESM significantly (*p* < 0.05) inhibited PLA_2_ activity, provoking inhibition comparable to that of prednisolone. The action of PLA_2_ on erythrocyte membrane creates leakage, causing haemoglobin to flow out into the medium (Oyekachukwu et al. 2017; Agatemor et al. 2019). The enzyme activity is thus directly related to the amount of haemoglobin in the medium; this is represented by the absorbance values (Table 2). Increase in inhibition of PLA_2_ reduces the amount of haemoglobin released. This suggests that the plant may suppress the synthesis of free fatty acids from membrane phospholipids and consequently deprive prostaglandin synthase of substrates for the production of prostaglandins. This inhibits the levels of pro-inflammatory mediators, such as prostaglandin E_2_ (PGE_2_). The crude extract was purified to remove all undesirable and inert components so as to increase effectiveness and lessen any taste, odour and colour (Barbosa-Pereira et al. 2013). The purification process enables a good level of PLA_2_-inhibitory activity to be obtained from relatively small concentrations of the purified extract. This was responsible for the increase in percentage inhibition of PLA_2_ activity at the lower concentrations of the partitions and fractions compared to the crude extract (Tables 2–4). PLA_2_-inhibitory activity of this plant could be attributed to alkaloids as chromone alkaloids isolated from *S. magnificum* possess anti-inflammatory activity against snake venom – which contains PLA_2_ (Houghton and Woldemariam 1993).

FTIR spectrum is an effective tool for differentiating, classifying and discriminating closely related plants and other organisms (Maitera and Chukkol 2016). It is perhaps the most powerful tool for identifying the types of chemical bonds (functional groups) present in compounds (Murugesh and Vino 2017). FTIR spectroscopic study revealed the presence of various functional groups in SMF1. The strong peak at 2924.18 cm^−1^ is ascribed to the C–H stretching of alkane compounds which are present in rare medicinal plants (Starlin et al. 2012). A strong absorption peak at 1033.88 cm^−1^ assigned to C–O of phenols is seen on the finger print region of the spectra, and phenols are known to modulate inflammation as they contain aromatic and alcohol groups. According to Owolabi et al. (2018), phenols modulate inflammation by reducing the synthesis of reactive oxygen and nitrogen species, hindering the activity of cyclooxygenase (COX) and inducible nitric oxide synthase (iNOS), suppressing inflammatory cytokines and chemokines synthesis and controlling NF-κβ signalling. The presence of phenols was confirmed in the phytochemical analysis of MESM and its partitions. Other medium and weak peaks observed from the spectra indicate the presence of amines, carboxylic acid, esters, aldehydes and alkenes. Carboxylic acids help in maintaining the cell membrane which is one of the underlining mechanisms by which plants inhibit PLA_2_ activity and inflammation (Krishna and Mohan 2017). Absent of a peak at 2260 cm^−1^ region indicates absence of cyanide group in SMF1 which shows the non-toxic nature of the plant (Ranjana and Vijay 2015).

GC-MS analysis is a breakthrough in analysing the phytoconstituents and elucidating the structure of compounds as they are very sensitive in detecting compounds as low as 1 ng (Saravanakumar et al. 2016). In this study, SMF1 was analysed by GC-MS and the major peak indicates *n*-hexadecanoic acid, ethyl ester (24.906%) which contain alkyl group as identified in FTIR. The functional group of esters and carboxylic acids were also revealed in the FTIR spectrum. Hexadecanoic acid, ethyl ester has previously been confirmed in a plant from the same family – *Borreria stachydea* (DC.) Hutch. & Dalziel (Rubiaceae) by Onoja and Odenigbo (2015). It acts as an antioxidant, antiandrogenic, nematicide, lubricant, haemolytic, hypocholesterolemic, flavour, pesticide and 5-α reductase inhibitor (Rajeswari et al. 2013). Hexadecanoic acid, ethyl ester also possesses anti-inflammatory property (Saeed et al. 2012; Echavarría et al. 2020). The anti-inflammatory activity of *n*-hexadecanoic was revealed from structure and kinetic study carried out by Aparna et al. (2012) to be due to its ability to inhibit PLA_2_ competitively. 14-Methyl pentadecanoic acid methyl ester (13.351%) detected in this study have also been confirmed in *Andrographis paniculata* (Burm. f.) Nees (Acanthaceae) (Mohanapriya et al. 2017) – a plant possessing PLA_2_-inhibitory activity – suggesting the involvement of this compound in mediating the PLA_2_-inhibitory activity of *S. magnificum* leaves. This study further revealed the presence of other compounds which may possess anti-inflammatory properties and other therapeutic importance.

## Conclusions

This study confirmed that MESM mediates its anti-inflammatory action *via* the inhibition of PLA_2_, thus substantiating its traditional use as anti-snake bite agent. This activity could be attributed to the compounds present in the plant of which hexadecanoic acid, ethyl ester and 3-ethylhexane, are the most abundant. Hexadecanoic acid, ethyl ester, which possesses anti-inflammatory properties, has been shown to act by inhibiting PLA_2_. The individual constituents can therefore be isolated and subjected to biological activities. Specifically, further research can be directed at examining the pharmacological activities of the constituents in the treatment of snake bites and inflammation-related disorders.
